# Molecular Characterization of the Clinical and Tumor Immune Microenvironment Signature of 5-methylcytosine-Related Regulators in non-small Cell Lung Cancer

**DOI:** 10.3389/fcell.2021.779367

**Published:** 2021-11-11

**Authors:** Taisheng Liu, Liyi Guo, Guihong Liu, Xiaoshan Hu, Xiaoning Li, Jinye Zhang, Zili Dai, Peng Yu, Ming Jiang, Jian Wang, Jian Zhang

**Affiliations:** ^1^ Department of Thoracic Surgery, Affiliated Cancer Hospital and Institute of Guangzhou Medical University, Guangzhou, China; ^2^ The Sixth People’s Hospital of Huizhou City, Huiyang Hospital Affiliated to Southern Medical University, Huizhou, China; ^3^ Department of Radiation Oncology, DongGuan Tungwah Hospital, Dongguan, China; ^4^ Department of Internal Medicine of Oncology, Affiliated Cancer Hospital and Institute of Guangzhou Medical University, Guangzhou, China; ^5^ Department of Radiation Oncology, Affiliated Cancer Hospital and Institute of Guangzhou Medical University, State Key Laboratory of Respiratory Diseases, Guangzhou Institute of Respiratory Disease, Guangzhou, China; ^6^ Department of Breast Surgery, Affiliated Cancer Hospital and Institute of Guangzhou Medical University, Guangzhou, China

**Keywords:** lung adenocarcinoma, 5mC, tumour microenvironment, immunotherapy, mutation burden

## Abstract

**Background:** DNA methylation is an important epigenetic modification, among which 5-methylcytosine methylation (5mC) is generally associated with tumorigenesis. Nonetheless, the potential roles of 5mC regulators in the tumor microenvironment (TME) remain unclear.

**Methods:** The 5mC modification patterns of 1,374 lung adenocarcinoma samples were analyzed systematically. The correlation between the 5mC modification and tumor microenvironment cell infiltration was further assessed. The 5mCscore was developed to evaluate tumor mutation burden, immune check-point inhibitor response, and the clinical prognosis of individual tumors.

**Results:** Three 5mC modification patterns were established based on the clinical characteristics of 21 5mC regulators. According to the differential expression of 5mC regulators, three distinct 5mC gene cluster were also identified, which showed distinct TME immune cell infiltration patterns and clinical prognoses. The 5mCscore was constructed to evaluate the tumor mutation burden, immune check-point inhibitor response, and prognosis characteristics. We found that patients with a low 5mCscore had significant immune cell infiltration and increased clinical benefit.

**Conclusion:** This study indicated that the 5mC modification is involved in regulating TME infiltration remodeling. Targeting 5mC modification regulators might be a novel strategy to treat lung cancer.

## Introduction

Lung cancer is the primary cause of cancer-related deaths worldwide (Siegel et al., 2020) (NSCLC), accounting approximately for 85% of newly diagnosed lung cancer cases, is classified into lung adenocarcinoma (LUAD) and lung squamous carcinoma (LUSC) (Curran et al., 2011). For unresectable advanced NSCLC, a combination of radiotherapy and chemotherapy has been the most common first-line treatment (Yoda et al., 2019), and impressive clinical success has been observed using targeted therapies (Treat, 2005; Yuan et al., 2019; Alexander et al., 2020). Unfortunately, most NSCLC patients will suffer the relapse within 1 year (Fountzilas et al., 2021). Thus, understanding the mechanism and identifying novel targets to treat NSCLC remain an urgent clinical need.

Immunotherapies represent a promising advance in cancer treatment (Lussier et al., 2021). The immune checkpoint inhibitors (ICI), including programmed death-ligand 1 (PD-L1), programmed cell death 1 (PD-1), and cytotoxic T-lymphocyte antigen-4 (CTLA-4), combined with chemoradiotherapy, have been approved or are being widely evaluated in clinical trials (Grant et al., 2021). However, targeting PD-1 or PD-L1 has demonstrated durable efficacy only in a subset of patients with NSCLC (Jazieh et al., 2021). Thus, it is important to determine the underlying mechanisms with the aim of improving the curative effect.

DNA methylation is an epigenetic modification that is associated with regulating cell differentiation and tissue development (Smith and Meissner, 2013; Slieker et al., 2015). Dysregulation of DNA methylation patterns are important characteristics of several diseases, including cancers (Li et al., 2013; Božić et al., 2021; Cristall et al., 2021; Miyakuni et al., 2021). 5-Methylcytosine (5mC), a type of DNA methylation, was firstly reported by Wyatt, (1951). DNA 5mC methylation is the classic epigenetic process, which is controlled by “writers” (DNA methyltransferases), “erasers” (DNA methyltransferases), and “readers” (Ito et al., 2011; Du et al., 2015; Lio et al., 2020). With the discovery of 5mC regulators, recent studies suggested that DNA cytosine modifications may act as epigenetic markers in tumorigenesis (Wu and Zhang, 2010; Cavalcante et al., 2020; Jiang, 2020; Mo et al., 2020) and can regulate tumor microenvironment (TME) infiltrating cells (Chen et al., 2020; Zhao et al., 2021; Onodera et al., 2021). However, the comprehensive roles of TME cell infiltration directed by 5mC regulators in NSCLC remain unclear.

In this study, we evaluated 5mC methylation patterns comprehensively by analyzing genomic information of 1374 LUAD samples, and correlated the 5mC methylation pattern with the characteristics of TME cell infiltration. We identified three 5mC methylation patterns, and revealed that 5mC methylation mediation of TME cell infiltration characteristics was closely associated with the immune response phenotype, indicating the 5mC methylation played an important role in modifying TME characteristics. Furthermore, the 5mCscore could be applied as a promising biomarker to predict immune response and clinical outcome in NSCLC.

## Materials and Methods

### Dataset Acquisition and Processing

Supplementary Figure S1 shows the workflow of the this study. mRNA expression with clinical and survival information were downloaded from Gene Expression Omnibus (GEO) and GDC data portal. Patients without clinical survival information were excluded. Five eligible lung adenocarcinoma cohorts (GSE19188, GSE31210, GSE37745, GSE50081, and TCGA-LUAD [lung adenocarcinoma data from The Cancer genome Atlas (TGCA)]) were included for further analysis (Supplementary Table S1). For background correction and normalization, the Robust Multichip Average algorithm was used to uniformly process the raw. CEL files of the four GEO datasets (Gautier et al., 2004). Next, a GEO meta-cohort were created by merging the GEO datasets using the R sva package (Leek et al., 2012).

Twenty-one 5mc regulators, including three writers (DNA methyltransferase 1 (DNMT1), DNA methyltransferase 3 Alpha (DNMT3A), DNA methyltransferase 3 beta (DNMT3B)), three erasers (tet methylcytosine dioxygenase 1 (TET1), tet methylcytosine dioxygenase 2 (TET2), tet methylcytosine dioxygenase 3 (TET3)), and 15 readers (methyl-cpg binding domain protein 1 (MBD1), methyl-cpg binding domain protein 2 (MBD2), methyl-cpg binding domain protein 3 (MBD3), methyl-cpg binding domain protein 4 (MBD4), methyl-cpg binding protein 2 (MECP2), nei like dna glycosylase 1 (NEIL1), *n*th like dna glycosylase 1 (NTHL1), single-strand-selective monofunctional uracil-dna glycosylase 1 (SMUG1), thymine dna glycosylase (TDG), ubiquitin like with phd and ring finger domains 1 (UHRF1), ubiquitin like with phd and ring finger domains 2 (UHRF2), uracil dna glycosylase (UNG), zinc finger and btb domain containing 33 (ZBTB33), zinc finger and btb domain containing 34 (ZBTB34), zinc finger and btb domain containing 4 (ZBTB4)) (Chen et al., 2020), and 23 tumor immune related cells from published studies (Zhang et al., 2020a; Zhao et al., 2021), were included for analysis. The transcriptomics data, single nucleotide variant (SNV), copy number variation (CNV), and 5mC phenotypic data were collected using the UCSC Xena database (https://xenabrowser.net) and the GDC data portal.

### Unsupervised Clustering of 21 5mC Regulators

To identify 5mC regulator-mediated modification sub-clusters, unsupervised consensus clustering was used to cluster tumor samples into sub-clusters based on the expression levels of the 21 5mC regulators. To ensure the stability of the clusters, the parameters of clustering were as follows: number of repetitions = 1,000 bootstraps, clustering algorithm = k-means method, pFeature = 1.0, pItem = 0.8. The cluster with the most significant survival difference was included for further analysis.

### Gene Set Variation Analysis and Functional Annotation

To explore the biological behavior among the different 5mC modification patterns, their pathway scores were evaluated using gene set variation analysis (GSVA) using the R GSVA package (Hänzelmann et al., 2013), with the “c2. cp.kegg.v7.4. symbols” gene set as the background. Differential pathways were further screened using *p* < 0.05 in the R package limma.

### Estimation of the Tumor Microenvironment

To identify the TME cell infiltration in LUAD samples, the relative abundances of immune cells were quantified using the single-sample gene-set enrichment analysis (ssGSEA) algorithm. According to the method revealed by Charoentong et al. (2017b), various kinds of immune cells, including regulatory T cells, activated CD8^+^ T cells, dendritic cells, and B cells, were evaluated. The relative abundance of TME infiltrating cells in clinical samples was represented by the enrichment scores.

### Differentially Expressed Genes

To identify 5mC-related differentially expressed genes (DEGs), based on the expression levels of 21 5mC regulators, three distinct 5mC modification patterns were identified in the patients with LUAD. The empirical Bayesian approach of R package limma package was used for the difference analysis (Ritchie et al., 2015), which screened out 324 DEGs, 246 DEGs and 144 DEGs according to *p* < 0.001, *p* < 0.0005 and *p* < 0.0001. *p* < 0.0005 was most suitable for subsequent analysis.

### Construction of 5mC Gene Signatures

Considering the heterogeneity and complexity of tumors, and according to the method used by Zhang J. et al. (2020), the 5mCscore was developed to quantify the modification pattern of individual patients with LUAD based on the identified DEGs. A univariate Cox regression model was used for the prognostic analysis of each gene in the 5mC signatures. We obtained 103 genes related to prognosis from among the 246 DEGs, and then principal component analysis (PCA) was performed, scored as PCi_1_ and PCi2. This approach had advantage of focusing the score on the set with the largest block of well correlated (or anticorrelated) genes in the set, while down-weighting contributions from genes that do not track with other set members. The 5mC score of each patient was calculated as follows:

$$5mCscore=PCi_{1}+PCi_{2}$$

### Evaluation of Immune-Checkpoint Inhibitor Genomic and Clinical Information

To explore the application of the 5mC score to predict immune-checkpoint inhibitor (ICI) efficacy, the expression data and clinical annotations of the immunotherapeutic cohort of atezolizumab (IMvigor210 cohort) were downloaded from the website based on the Creative Commons 3.0 License (http://research-pub.Gene.com/imvigor210corebiologies) (Mariathasan et al., 2018).

### Statistical Analysis

Correlation coefficients between the expression of 5mC regulators and the TME immune infiltration cells was conducted using the Spearman method and distance correlation analysis. The Wilcoxon test was used to analyze the difference between two groups. The Kruskal–Wallis test and one-way analysis of variance (ANOVA) were used to analyze difference among three or more groups. The log-rank test and the Kaplan–Meier (KM) method were applied to evaluate the survival time. A statistical two-sided *p* value < 0.05 was considered as having significance. All data processing in this study was done using R 3.6.1 software.

## Results

### Genetic Variation and Expression Analysis of 5mC Methylation Regulators

**FIGURE 1:**
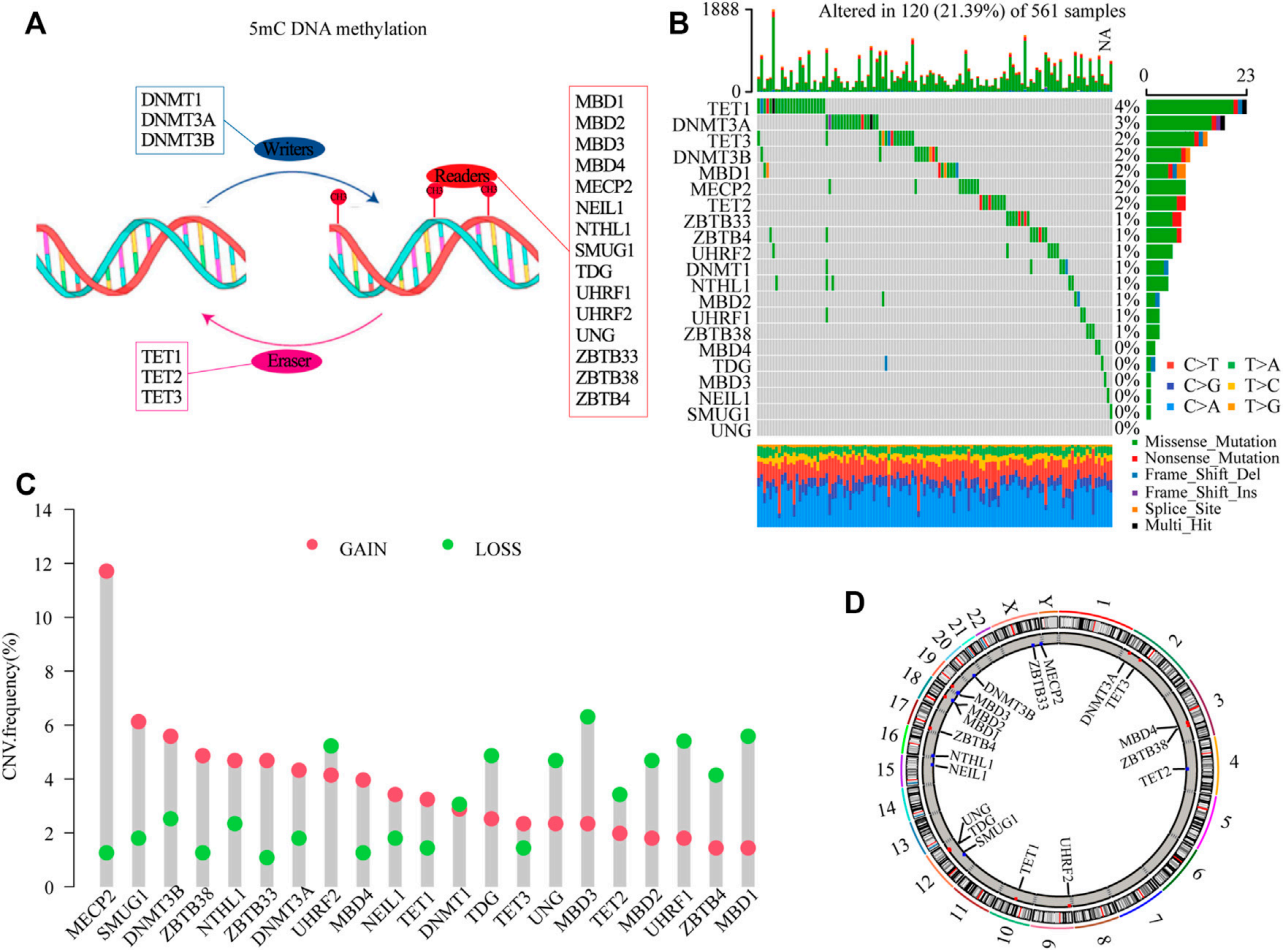
Genetic landscape and expression analysis of 5mC regulators in LUAD. **(A)** Schematic diagram of 5mC DNA methylation mediated by 21 5mC regulators. **(B)** The mutation frequency of 21 5mC regulators in the TCGA-LUAD cohort. The column indicates individual patients. The upper barplot shows the TMB, The number on the right indicates the mutation frequency. The right barplot shows the percentage of mutation type in each regulator. The stacked barplot shows the fraction of conversions. **(C)** The CNV variation frequency of 5mC regulators in the TCGA-LUAD cohort. The height of the column indicates the alteration frequency of the regulators. The green dot is the deletion frequency; The red dot is the amplification frequency. **(D)** The locations of CNV alterations of 5mC regulators in the TCGA-LUAD cohort. CNV, copy number variation; 5mC, 5-methylcytosine; LUAD, lung adenocarcinoma; TCGA, The Cancer Genome Atlas; TMB, tumor mutation burden.

According to the map described in Figure 1A, in this study, 21 5 mC methylation regulators (writers: DNMT1, DNMT3A and DNMT3B; erasers: TET1, TET2, TET3; readers: MBD1, MBD2, MBD3, MBD4, MECP2, NEIL1, NTHL1, SMUG1, TDG, UHRF1, UHRF2, UNG, ZBTB33, ZBTB38, and ZBTB4) were identified (Supplementary Table S2). To determine genetic alternations, we firstly evaluated the SNV variation frequency of the genes encoding the 21 5mC methylation regulators. As shown in Figure 1B, Among 561 LUAD samples, 21.39% of 5mC regulators had mutations. The main mutation type was missense_mutation. However, the mutation frequency of individual regulators only ranged from 0 to 4%. The CNV frequency of the 5 mC regulators showed that *MECP2*, *SMUG1*, *DNMT3B*, *ZBTB33*, and *NTHL1* had distinct CNV amplification, with frequencies of 11.71, 6.13, 5.58, 4.68, and 4.68%, respectively. *MBD3*, *UHRF1*, *MBD1*, *UHRF2*, and *TDG* had a CNV deletion, with frequencies of 6.30, 5.40, 5.58, 5.23, and 4.86%, respectively (Figure 1C and Supplementary Table S3). The distribution analysis of CNV alterations on 23 chromosomes showed that their distribution among the 21 5mC regulators was scattered and unorganized (Figure 1D). Survival analysis indicated that high expression of *DNMT3B*, *MDB2*, *MDB3*, *SMUG1*, *TDG*, *HURF1*, *UNG*, and *ZBTB38* were associated with poor survival of LUAD (*p* < 0.05); while, high expression of *MDB4*, *MECP2*, *NEIL1*, *TET2*, *UHRF2*, and *ZBTB4* were associated with better survival of LUAD (*p* < 0.05, Supplementary Figure S2 and Supplementary Table S4).

### Identification of 5mC Methylation-Related Phenotypes

**FIGURE 2:**
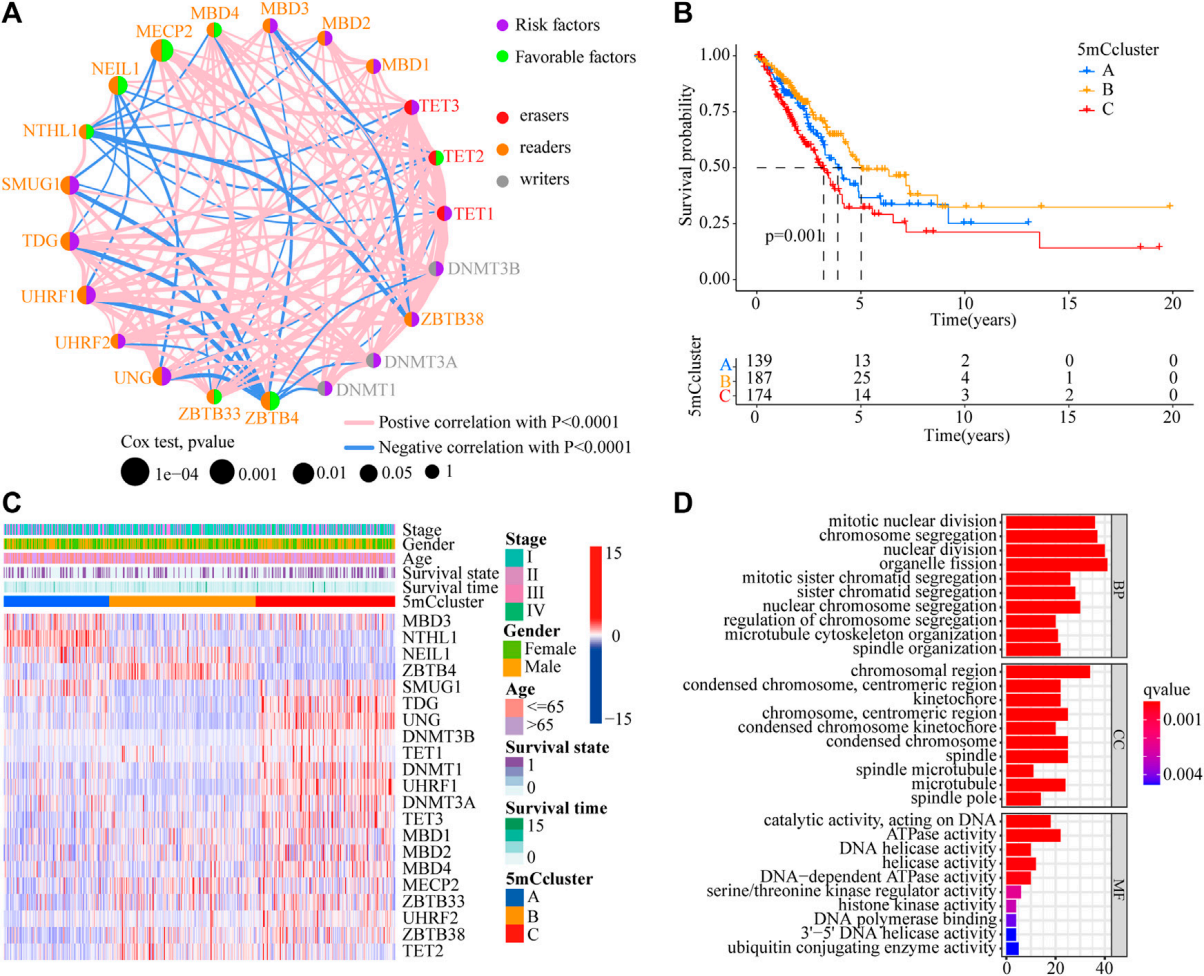
Prognostic and biological characteristics of 5mC modification patterns in LUAD. **(A)** The interaction among 5mC regulators in the TCGA-LUAD cohort. The circle size indicates the effect of each regulator on prognosis. Green dots indicate favorable factors for prognosis; Purple dots indicate risk factor for prognosis. The lines linking regulators indicate their interactions, and thickness show the correlation strength between the regulators. Negative correlations are marked in blue and positive correlation in red. **(B)** Survival analyses of 5mC modification patterns in the TCGA-LUAD cohort, including 500 cases in 5mC cluster A (*n* = 139), 5mC cluster B (*n* = 187), and 5mC cluster C (*n* = 174) (*p* < 0.0001, Log-rank test). **(C)** Cluster analysis of 21 5mC regulators among the three 5mC modification patterns. **(D)** Gene ontology (GO) analysis of 21 5mC regulators among the three 5mC modification patterns.

To determine the roles of interaction among 5 mC methylation regulators in LUAD, correlation analysis among the 21 5 mC regulators was performed, which showed that there was a strong positive correlation among most of the regulators (Supplementary Figure S3A and Supplementary Table S5). The prognostic values of the 21 5 mC regulators in LUAD were evaluated using a univariate Cox regression model (Supplementary Figure S3B). As shown in Figure 2A, *MDB4*, *MECP2*, *NEIL1*, *TET2*, *ZBTB4*, and *ZBTB33* were favorable factors for overall survival (OS), while *DNMT1*, *DNMT3A*, *DNMT3B*, *TET1*, *TET3*, *SMUG1*, *TDG*, *UHRF1*, *UHRF2*, *UNG*, and *ZBTB38* were risk factors for OS. Significant negative correlations were obtained for UHRF1 and DNMT1, TDG and DNMT3A, TDG and UNG, MECP2 and ZBTB33, MECP2 and TET2, and TET2 and UHRF2 (*p* < 0.001). On the other hand, several erasers and readers also showed significant negative correlations: NTHL1 and TET2, NTHL1 and TET3, and MBD3 and TET2 (*p* < 0.001) (Supplementary Tables S6–7). Using unsupervised clustering analysis, three distinct 5mC modification patterns were identified based on the expression of 21 5mC regulators (Supplementary Figure S4). Prognostic analysis of the three 5mC modification clusters revealed a particularly prominent survival advantage for the 5mC cluster-B modification pattern (Figure 2B and Supplementary Table S8; *p* = 0.001). The results showed that cross-talk among the 5mC modification regulators might be involved in the formation of the 5mC modification and in the characteristics of TME cell infiltration.

### Tumor Microenvironment Cell Infiltration Characteristics in the 5mC Methylation Clusters

**FIGURE 3:**
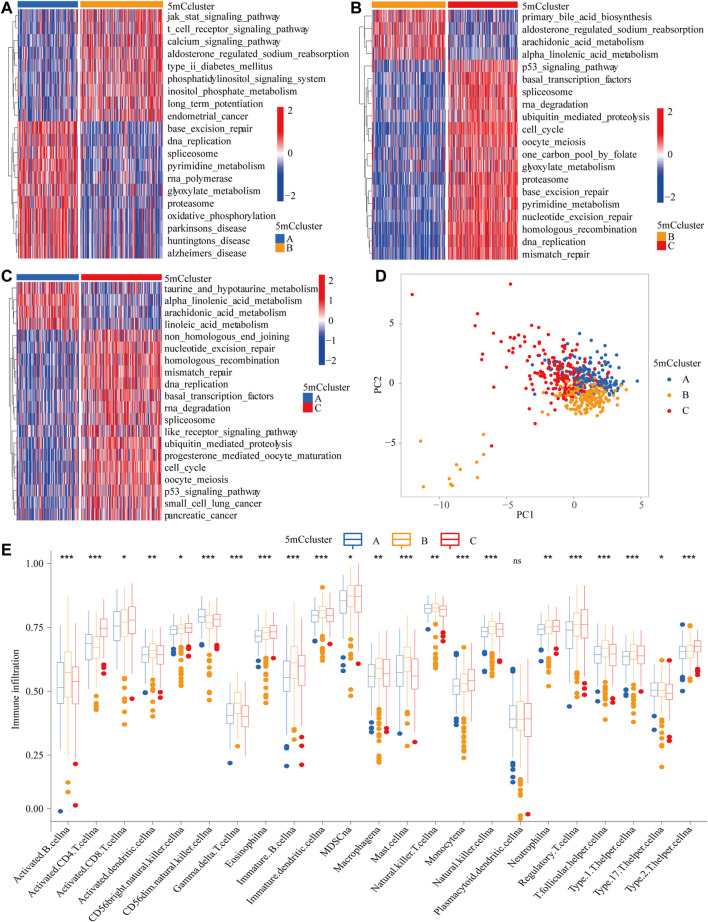
GSVA enrichment analysis and TME cell infiltration characteristics of 5mC modification patterns. **(A–C)** The states of biological pathways among the three 5mC modification patterns enriched by GSVA analysis The general biological processes are shown as a heatmap, red represents activated pathways and blue represents inactivated pathways. **(A)** 5 mC cluster A *vs* 5mC cluster B; **(B)** 5mC cluster B *vs* 5mC cluster C; **(B)** 5 mC cluster A *vs* 5mC cluster C. **(D)** Principal component analysis of the 5mC modification patterns. **(E)** The abundance of TME infiltrating cells in the 5mC modification patterns (*p < 0.05; **p < 0.01; ***p < 0.001; ns means not significant).

To identify the potential function of the differentially expressed 5mC regulators, cluster analysis was first performed. As shown in Figure 2C, the 21 5mC regulators had a distinct distribution among the three 5mC clusters. Gene ontology (GO) analysis was performed to identify the biological process (BP), cellular component (CC), and molecular function (MF) of the regulators. The aberrantly expressed 5mC regulators were mainly enriched for GO terms related to regulation of mitotic nuclear division, chromosome segregation, and nuclear division (BP); chromosomal region, condensed chromosome/centromeric region, and kinetochore (CC); and ATPase activity, DNA helicase activity, and helicase activity (MF) (Figure 2D). To further identify the potential behaviors, GSVA enrichment analysis was performed, as shown in Supplementary Table S9. To further exlpore unsupervised consensus clustering of all tumor samples for the molecular classification of LUAD. The optimal number of clusters was determined by the K value. After assessing relative changes in the area under the cumulative distribution function curve and consensus matrix heatmap, we selected a three-cluster solution (K = 3), which showed no appreciable increase in the area under the cumulative distribution function curve (Supplementary Figure S4). 5mC cluster A was markedly enriched in damage repair-related pathways, such as base excision repair, DNA replication, spliceosome, and RNA polymerase. 5mC cluster B was prominently related to immune activation-related pathways, such as the JAK-STAT signaling pathway, the T cell receptor signaling pathway, and the calcium signaling pathway. 5mC cluster C was mostly associated with carcinogenic activation and damage repair pathways, such as, the p53 signaling pathway, basal transcription factors, spliceosome, RNA degradation, DNA replication, base excision repair, homologous recombination, DNA replication, and mismatch repair (Figures 3A–C). Based on the expression levels of these 21 5mC regulators, the three 5mC modification patterns could be partially differentiated using PCA (Figure 3D). TME cell infiltration analysis showed 5mC cluster B was associated with activated B cells, activated dendritic cells, mast cells, natural killer T cells, and neutrophils (Figure 3E and Supplementary Table S10, *p* < 0.001). 5mC cluster C was remarkably rich in immune cell infiltration including myeloid-derived suppressor cells (MDSCs), regulatory T cells, type 1 T helper cells, type 2 T helper cells, and type 17 T helper cell (Figure 3E and Supplementary Table S10, *p* < 0.001). Prognosis analysis showed that patients with different 5mC modification patterns also had a matching survival advantage (Figure 2B, *p* = 0.001). Based on the above results, cluster A, characterized by innate immune cell infiltration, was defined as an immune-excluded phenotype; cluster B, characterized by adaptive immune cell infiltration and immune activation, was defined as an immune-inflamed phenotype; and cluster C, characterized by the inhibition of immunity, was defined as an immune-desert phenotype.

### Identification of 5mC Methylation Gene Signature

**FIGURE 4:**
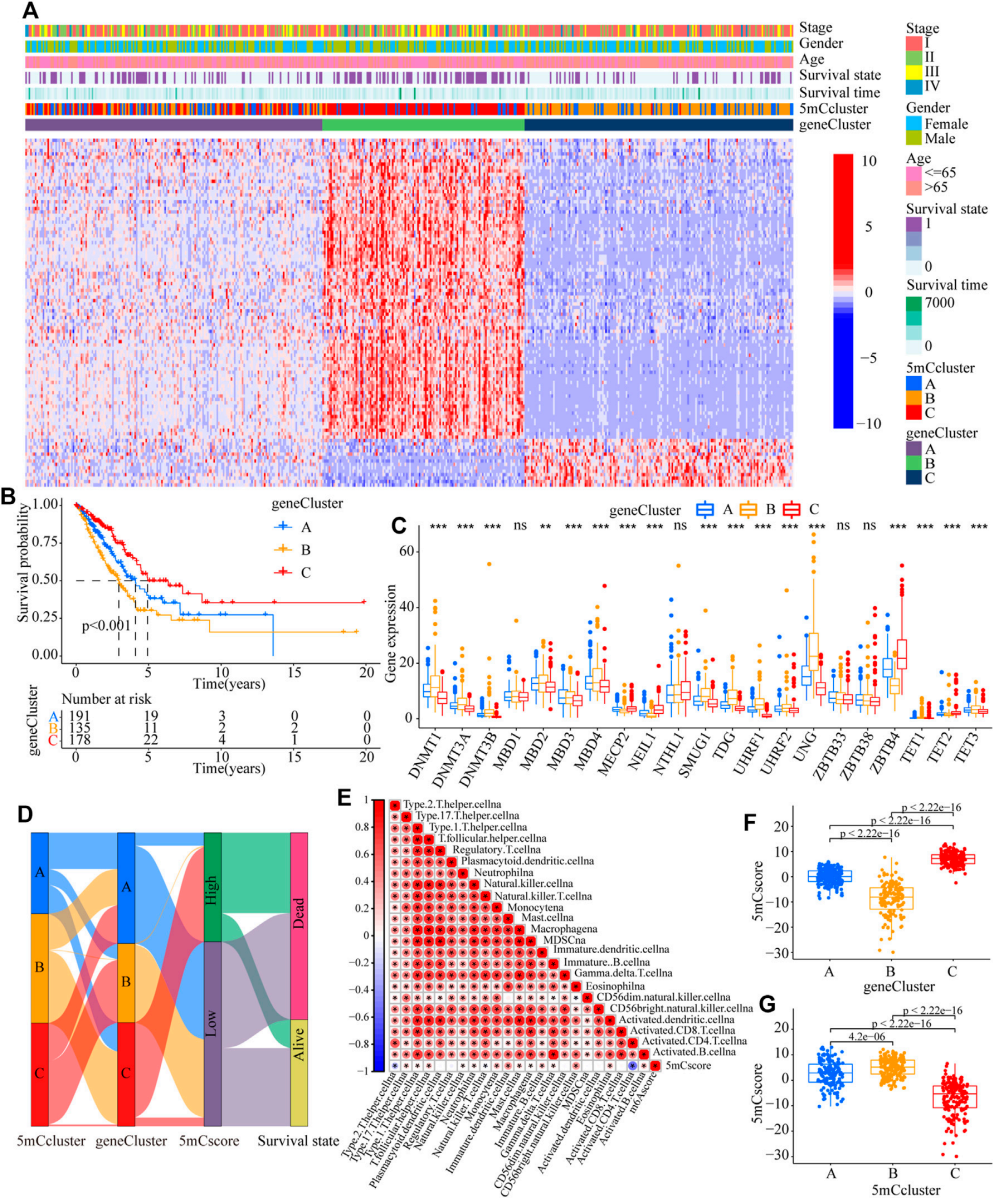
Construction of 5mC gene signatures. **(A)** Unsupervised clustering of overlapping 5mC phenotype-related genes in the TCGA-LUAD cohort to classify patients into different genomic subtypes, termed as 5mC gene cluster **(A–C)**, respectively. The gene clusters, 5mC clusters, tumor stage, survival status, sex, and age were used as patient annotations. **(B)** Overall survival of patients with the three 5mC modification genomic clusters in the TCGA-LUAD cohort, including 504 cases in 5mC gene cluster A (*n* = 191), 5mC gene cluster B (*n* = 135), and 5mC gene cluster C (*n* = 17) (*p* < 0.0001, Log-rank test). **(C)** The expression of 21 5mC regulators in the three gene clusters (*p < 0.05; **p < 0.01; ***p < 0.001; ns means not significant). **(D)** Alluvial diagram showing the changes in 5mC clusters, 5mC gene cluster, 5mCscore, and survival. **(E)** Correlations between the 5mCscore and the known gene signatures in the TCGA-LUAD cohort using Spearman analysis. Negative correlations are marked in blue and positive correlation in red. **(F)** Differences in the 5mCscore among three gene clusters in the TCGA-LUAD cohort (***p < 0.001, Kruskal-Wallis test). **(G)** Differences in the 5mCscore among three the 5mC modification patterns in the TCGA-LUAD cohort (***p < 0.001, Kruskal-Wallis test).

To further identify the potential function of each m5C modification pattern, we determined 246 m5C phenotype-related DEGs (Supplementary Table S11). GO analysis showed that the 246 DEGs were associated with cell cycle, RNA transport, spliceosome, DNA replication, base excision, and human T-cell leukemia virus 1 infection (Supplementary Figure S5A and Supplementary Table S12). Kyoto Encyclopedia of Genes and Genomes (KEGG) analysis indicated that the 5mC gene clusters were involved in DNA transcription and translation (Supplementary Figure S5B and Supplementary Table S13). To further determine the potential regulation mechanism, unsupervised clustering analyses was performed to identify the genomic subtypes based on the 103 prognostic genes from the 246 5mC phenotype-related DEGs. The results showed that three distinct 5 mC genomic phenotypes (5mC gene Cluster A–C) could be identified (Figure 4A and Supplementary Table S5C–J). These results indicated that the 5mC methylation modification patterns did exist in LUAD and three distinct 5mC gene clusters were characterized by different signature genes. Cluster analysis showed that 178 of 504 patients with LUAD were clustered in 5mC gene cluster C, which was associated with better prognosis. Patients with LUAD with 5mC gene cluster B (*n* = 135) had poorer prognosis. 5mC gene cluster A, with 191 patients clustered, had an intermediate prognosis (Figure 4B, *p* < 0.001). The expression levels of the 5mC regulators among the 5mC gene clusters were distinctly different (Figure 4C).

### Clinical Characteristics of 5mCscore Phenotypes

To better explore the pattern of 5mC modification in individual patients, based on the 5mC phenotype-related genes (Supplementary Table S14), the 5mCscore was used to quantify the 5mC modification patterns of individual patients with LUAD. An alluvial diagram was applied to clarify the attributed changes of the LUAD patients. As shown in Figure 4D, the 5mC modification patterns clusters were almost consistent with the 5mC gene clusters, i.e., the 5mC gene cluster B group patients mainly had a low 5mCscore, which was associated with poor survival. To determine the roles of 5mC-related phenotypes in immune regulation, correlation analysis showed that the 5mCscore was associated positively with most TME infiltrating cells (Figure 4E). The Kruskal–Wallis test revealed there was a significant difference in the 5mCscore among the 5mC gene clusters. 5mC gene cluster C showed the highest median 5mCscore, while 5mC gene cluster B had the lowest median 5mCscore, which indicated that a high 5mCscore was closely associated with immune activation-related signatures, whereas a low 5mCscore was associated with immune inactivation-related signatures (Figure 4F, *p* < 0.001). More importantly, compared with the other clusters, 5mC modification cluster C presented the lowest median 5mCscore, and 5mC modification cluster B showed the highest 5mCscore (Figure 4G, *p* < 0.001). These results indicated that a high 5mCscore correlated significantly with immune-activation and the 5mCscore could be used to identify the 5mC modification patterns in LUAD, and further assess the characteristics of TME cell infiltration of individual tumors.

**FIGURE 5:**
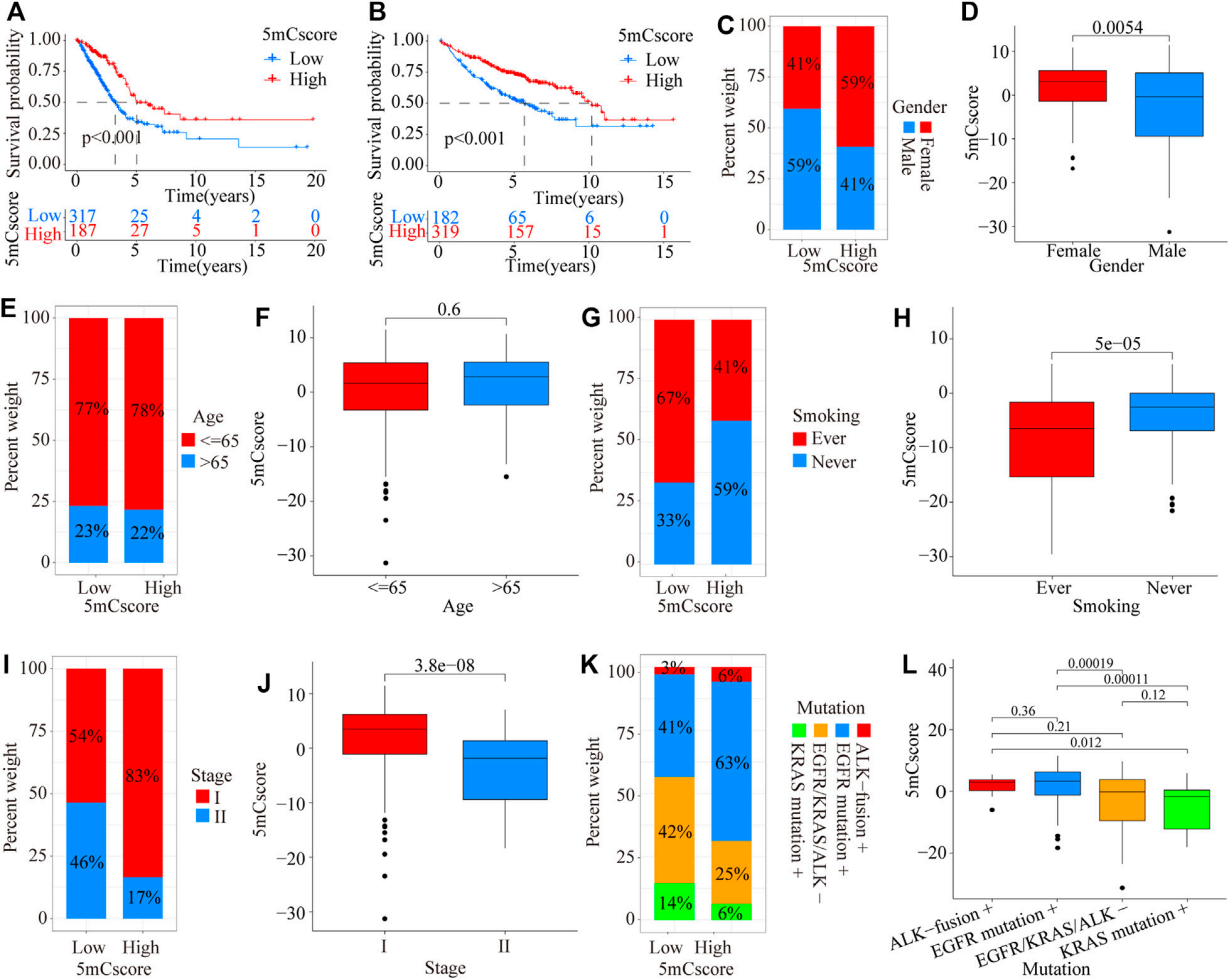
Prognostic and genetic characteristics between high and low 5mCscore groups. **(A)** Survival analysis of the 5mCscore in the TCGA-LUAD cohort (*p* < 0.0001, Log-rank test). **(B)** Survival analysis of the 5mCscore in the GEO-meta cohort (*p* < 0.0001, Log-rank test). **(C)** Sex proportion between the high- and low-5mCscore groups. **(D)** The 5mCscore difference between females and males. **(E)** Age proportion between the high- and low-5mCscore groups. **(F)** The 5mCscore difference between age (≤65) and age (>65). **(G)** Smoking status proportion between the high- and low-5mCscore groups. **(H)** The 5mCscore difference between smoking status (ever) and smoking status (never). **(I)** Clinical stage status proportion between the high- and low-5mCscore groups. **(J)** The 5mCscore difference between stage I and stage II. **(K)** The 5mCscore difference between genetic mutations (−) and genetic mutations (+). **(L)** Genetic mutations status proportion between high- and low-5mCscore groups.

To further validate the value of the 5mCscore, patients in the TCGA cohort were divided into low or high 5mCscore groups. Prognosis analysis showed that patients with a high 5mCscore showed a better survival benefit (Figure 5A, *p* < 0.001). Four GEO datasets (GSE19188, GSE31210, GSE37745, and GSE50081, Supplementary Table S1) were integrated into one meta-cohort. Survival analysis in the GEO meta-cohort also identified that a high 5mCscore was linked to a better clinical outcome (Figure 5B, *p* < 0.001). These results indicated that the 5mCscore could act as an independent prognostic biomarker to evaluate patient outcomes. To explore the effect of clinical characteristics on the 5mCscore, the subgroups of clinical characteristics were further analyzed. A significant distribution difference of a high 5mCscore was observed for gender (59% in female *vs* 41% in male, *p* = 0.0054; Figures 5C,D), smoking status (41% *vs* 67% for ever smoking, P = 5e-05; Figures 5G,H), stage I–II (83% *vs* 54% for stage I, *p* = 3.8e-08; Figures 5I,J), and genetic mutations (63% *vs* 41% for EGFR mutations, *p* = 0.00019; 25% *vs* 42% in EGFR/KRAS/ALK mutations, *p* < 0.001; Figures 5K-L). However, there were no 5mCscore differences between age (≤65) and age (>65) (Figures 5E,F, *p* = 0.6). To assess the value of clinical characteristics, patients in the TCGA-LUAD cohort were further stratified by age (≤65/> 65), sex (female/male), T stage (T1–2/T3–4), N stage (N0–1/N2–3), M stage (M0/M1), and clinical stage (I–II/III–IV). We found that the clinical characteristics, particularly T1–2, N0–1, M0, and I–II clinical stages, could be clearly divided into high- and low-risk subgroups (Supplementary Figure S6). These results indicated that multiple clinical characteristics can have an effect on the 5mCscore, which led to the heterogeneity of 5mC regulators in LUAD.

### The Potential of the 5mCscore to Predict the Response to anti-PD-L1 Immunotherapy

**FIGURE 6:**
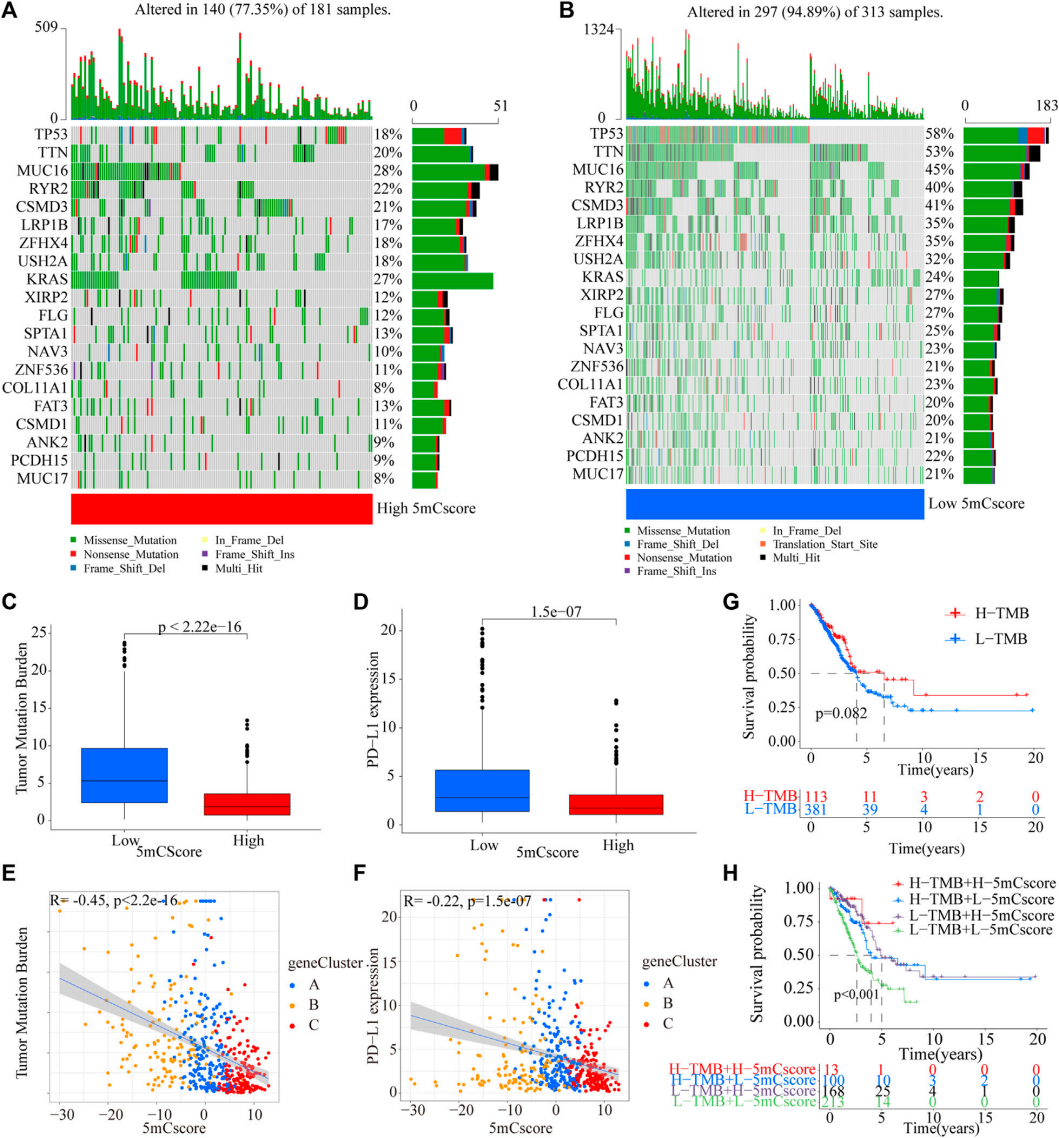
Characteristics of 5mCscore in the TCGA molecular subtypes and tumor somatic mutations. **(A,B)** Waterfall plot of tumor somatic mutations established by those with a high 5mCscore **(A)** and a low 5mCscore **(B)**. Each column represents individual patients. The upper barplot shows the TMB, the number on the right indicates the mutation frequency in each gene. The right barplot shows the proportion of each variant type. **(C)** Tumor somatic mutation between high 5mCscore and low 5mCscore groups. **(D)** PD-L1 expression difference between high 5mCscore and low 5mCscore groups. **(E)** The correlation analysis between tumor somatic mutation and the 5mCscore. **(F)** The correlation analysis between PD-L1 expression and the 5mCscore. **(G)** Survival analysis of tumor somatic mutations in the TCGA-LUAD cohort (*p* < 0.0001, Log-rank test). **(H)** Survival analyses for patients stratified by both the 5mCscore and the tumor somatic mutation burden using Kaplan–Meier curves (*p* < 0.0001, Log-rank test).

The above analyses demonstrated the impact of 5mCscore regulators on the TME, as well as on the prognosis in patients with LUAD. The genetic characteristics of the patients in different 5mCscore groups were further explored. As shown in Figures 6A,B and Supplementary Table S15, the somatic mutation landscapes in the high and low 5mCscore groups had a distinct difference. The mutation frequency was 77.35% in the high 5mCscore group and 94.89% in the low 5mCscore group. Specifically, except for *KRAS*, *TP53* (18% *vs* 58%), *TTN* (20% *vs* 53%), *MUC16* (28% *vs* 45%), and *RYR2* (22% *vs* 40%) had important differences between the high and low 5mCscore groups (Figure 6B). Besides, patients with a low 5mCscore showed a significantly higher tumor mutation burden (TMB) and PD-L1 expression than patients with a high 5mCscore (Figures 6C,D and Supplementary Table S16). 5mC gene cluster C showed lower PD-L1 expression and a lower TMB than 5mC gene cluster B. Correlation analysis further identified that the TMB and PD-L1 expression were related negatively with the 5 mCscore (Figures 6E,F, *p* < 0.001). These results revealed a significant association between the 5mCscore and the TMB and PD-L1 expression. These factors are important parameters in the assessment of immunotherapy outcomes. However, the survival analysis associated with the TMB found that there was no difference between the high and low TMB groups (Figure 6G, *p* = 0.082). Next, the crosstalk between the 5mCscore and TMB in terms of patient survival was investigated. The high 5mCscore and high TMB group had better survival than the low 5mCscore and high TMB group. The low 5mCscore and low TMB group was associated with poorer survival relative to those with a high 5mCscore and low TMB (Figure 6H, *p* < 0.001).

**FIGURE 7:**
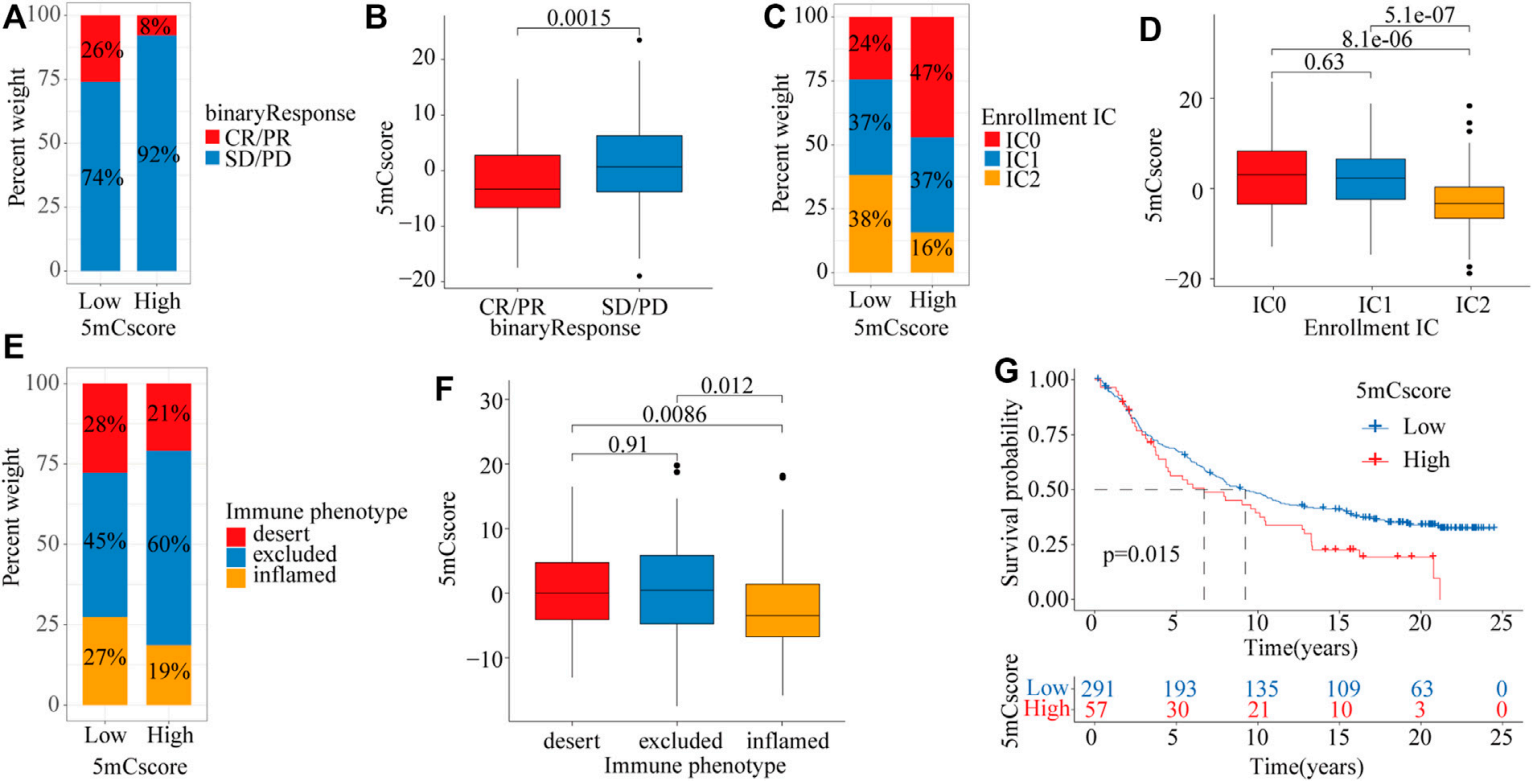
The role of the 5mCscore in anti-PD-L1 immunotherapy. **(A)** The proportion of patients with a response to ICI in the low or high 5mCscore groups. Responder/Nonresponder: 26%/74% in the low 5mCscore groups and 8%/92% in the high 5mCscore groups. **(B)** 5mCscore differences between responders and nonresponders. **(C)** IC infiltration proportion between high 5mCscore and low 5mCscore groups. **(D)** 5mCscore differences between different IC subgroups. **(E)** Immune phenotype proportion between high 5mCscore and low 5mCscore groups. **(F)** 5mCscore differences among the immune-desert phenotype, immune-excluded phenotype, and immune-inflamed phenotype. **(G)** Survival analyses for low (*n* = 291) and high (*n* = 57) 5mCscore patient groups in the anti-PD-L1 immunotherapy cohort using Kaplan–Meier curves (IMvigor210 cohort; *p* = 0.015, Log-rank test). CR, complete response; IC, immune cell; ICI, immune check-point inhibitor; PD, progressive disease; PR, partial response; SD, stable disease.

To explore the potential roles of the 5mCscore in clinical immune therapy of lung cancer, we investigated whether the 5mCscore could predict patients’ response to PD-L1 (atezolizumab) therapy based on the PD-L1 immunotherapy cohort (IMvigor210). Compared with those with a high 5mCscore, patients with a low 5mCscore had significant therapeutic advantages and clinical responses to anti-PD-L1 immunotherapy (Figures 7A,B and Supplementary Figure S7A−B, *p* = 0.0015). The low 5mCscore group had a higher immune cells 2 (IC2) score (38% *vs* 16%) and a lower tumor cells 2+ (TC2+) score (77% *vs* 96%) than the high 5mCscore group, 5mCscore was significantly associated with the enrollment ICs and suppression of TCs (Figures 7C,D and Supplementary Figure S7C−D). These results identified that the 5mCscore played a non-negligible role in regulating TME immune cell infiltration. We further investigated different immune phenotypes among the high and low 5mCscore groups and found that a higher 5mCscore was markedly associated with exclusion and desert immune phenotypes, in which an antitumor effect is difficult to exert using ICI therapy (Figures 7E,F). Patients with a low 5mCscore exhibited significant clinical benefits and a markedly prolonged survival (Figure 7G, *p* = 0.015). These results clarified that 5mC modification patterns are significantly associated with immune phenotypes and PD-L1 expression, and that the 5mCscore could be a prominent biomarker to predict the response to ICI therapy.

## Discussion

DNA 5mC methylation is a dynamic and reversible post-transcriptional modification regulated by 5mC related regulators (Mayer et al., 2000; Oswald et al., 2000; Wu et al., 2020). Recent research highlighted the biological importance of 5mC modification on immune cell infiltration and tumor suppression (Schübeler, 2015; Dor and Cedar, 2018; Weng et al., 2021). However, most studies focused only on a single TME cell type or one 5mC related regulator, and the comprehensive roles of 5mC regulators on TME infiltration characteristics are not fully elaborated. Thus, further clarification of the potential roles of 5mC modification patterns in the infiltration of TME cells will raise our awareness of the effects of the heterogeneity and complexity of the TME on the response to ICI therapy and provide a novel biomarkers to evaluate the ICI response and predict prognosis.

Herein, three distinct 5mC methylation modification patterns were identified based on 21 5mC regulators. The patterns had significantly distinct TME cell infiltration characteristics. Based on the identified 246 5mC phenotype-related DEGs, three genomic clusters of 5mC-related genes were further identified, which were also validated for their association with transcription modification and immune infiltration. Recent studies had shown that DNA methylation can be involved in the maintenance and reinforcement of T cell exhaustion gene signatures (Pauken et al., 2016; Gate et al., 2018). In murine antigen-specific CD8 T cells, DNMT3A-mediated methylation impaired T cell expansion and led to immune cell exhaustion under treatment with anti-PD-1 *via* repression the expression of key genes (Ghoneim et al., 2017). By contrast, in the context of T cell exhaustion, the involvement of DNA methylation in the reprogramming of the T cells has also been reported (Araki et al., 2013), such as demethylation of the *PD1* promoter resulting in permanent CD8+T cell exhaustion. Uhrf1-mediated tnf-α gene methylation controlled proinflammatory macrophages in experimental colitis resembling inflammatory bowel disease (Qi et al., 2019). In breast cancer, ZBTB33 subcellular partitioning functionally linked LC3A/B, the tumor microenvironment, and cancer survival (Singhal et al., 2021). These results indicated that the 5mC modification is intimately involved in shaping TME landscapes.

Epigenetic alterations are associated extensively with the immune response and tumore evasion. A DNA methylation signature (the EPIMMUNE signature) has been identified as an epigenetic biomarker of the response to ICI. The multicenter and retrospective analysis revealed that the EPIMMUNE signature could predict the response to anti-PD-1 treatment in non-small-cell lung cancer (Seremet et al., 2016). In metastatic melanoma treated with CTLA-4 blockers, responders and non-responders to ICI had a differential DNA methylation pattern (Chida et al., 2021). To better understand the individual heterogeneity of TME-meditated 5mC modification patterns, the 5mCscore was established to assess the 5mC modification pattern of individuals with LUAD. 5mC gene cluster C, characterized by an immune inflamed phenotype, exhibited a higher 5mCscore, and 5mC gene cluster B, characterized by an immune excluded phenotype, had a lower 5mCscore. These results revealed the 5mCscore was a useful biomarker to comprehensively assess individual tumor 5mC modification patterns, which could be used to evaluate TME immune cell infiltration patterns. Prognosis analyses also identified that the 5mCscore was an independent prognostic biomarker in LUAD.

Alterations in 5mC regulatory genes might also be associated with variations in LUAD. In this study, we identified twenty driver genes, including *TP53*, *TTN*, *MUC16*, *RYR2*, and CSMD3. Moreover, variations in *KRAS* were associated significantly with alterations in 5mC regulatory genes. As an oncogene, *KRAS* mutations were reported frequently in a variety of tumors, including colorectal cancer (Prior et al., 2012), pancreatic cancer (Arner et al., 2019), and bladder cancer (Santha et al., 2020). Recent studies identified that *KRAS* might have a critical role in the immunoregulation of NSCLC (Li et al., 2021; Wang et al., 2021). Our data also revealed that the 5mCscore had a markedly negative correlation with PD-L1 expression and the TMB. The 5mCscore integrating the TMB could be the more effective biomarker to predict ICI response. We also identified the predictive value of the 5mCscore in the IMvigor210 cohort. The 5mCscore between non-responders and responders was significantly different. These results provided new insights to clarify different tumor immune phenotypes and improve the clinical response to ICI therapy.

## Conclusion

In summary, we comprehensively analyzed the potential mechanisms of 5mC methylation modification during the regulation of the TME. 5mC modification patterns contributed to the heterogeneity and complexity of the TME in LUAD, which was significantly associated with TMB, PD-L1 expression, and immune phenotypes. 5mCscore could act as a biomarker to predict a patient’s response to ICI therapy.

## Data Availability

The datasets presented in this study can be found in online repositories. The names of the repository/repositories and accession number(s) can be found in the article/Supplementary Material.

## Abbreviations

5mC: 5-methylcytosine methylation; TME: tumor microenvironment; NSCLC: (Siegel et al., 2020); LUAD: lung adenocarcinoma; LUSC: lung squamous carcinoma; ICI: The immune checkpoint inhibitors; PD-L1: programmed death-ligand 1; PD-1: programmed cell death 1; CTLA-4: cytotoxic T-lymphocyte antigen-4; GEO: Gene Expression Omnibus; SNV: single nucleotide variant; CNV: copy number variation; GSVA: gene set variation analysis; ssGSEA: single-sample gene-set enrichment analysis; DEGs: differentially expressed genes; ANOVA: one-way analysis of variance; KM: Kaplan–Meier; TMB: tumor mutation burden; GO: Gene ontology; BP: biological process; CC: cellular component; MF: molecular function; MDSCs: myeloid-derived suppressor cells.
