# Supplementary figures and images for: Molecular Characterization of the Clinical and Tumor Immune Microenvironment Signature of 5-methylcytosine-Related Regulators in non-small Cell Lung Cancer
Source: Front Cell Dev Biol. 2021 Nov 11;9:779367. doi: 10.3389/fcell.2021.779367 (PMC8632062; doi:10.3389/fcell.2021.779367)

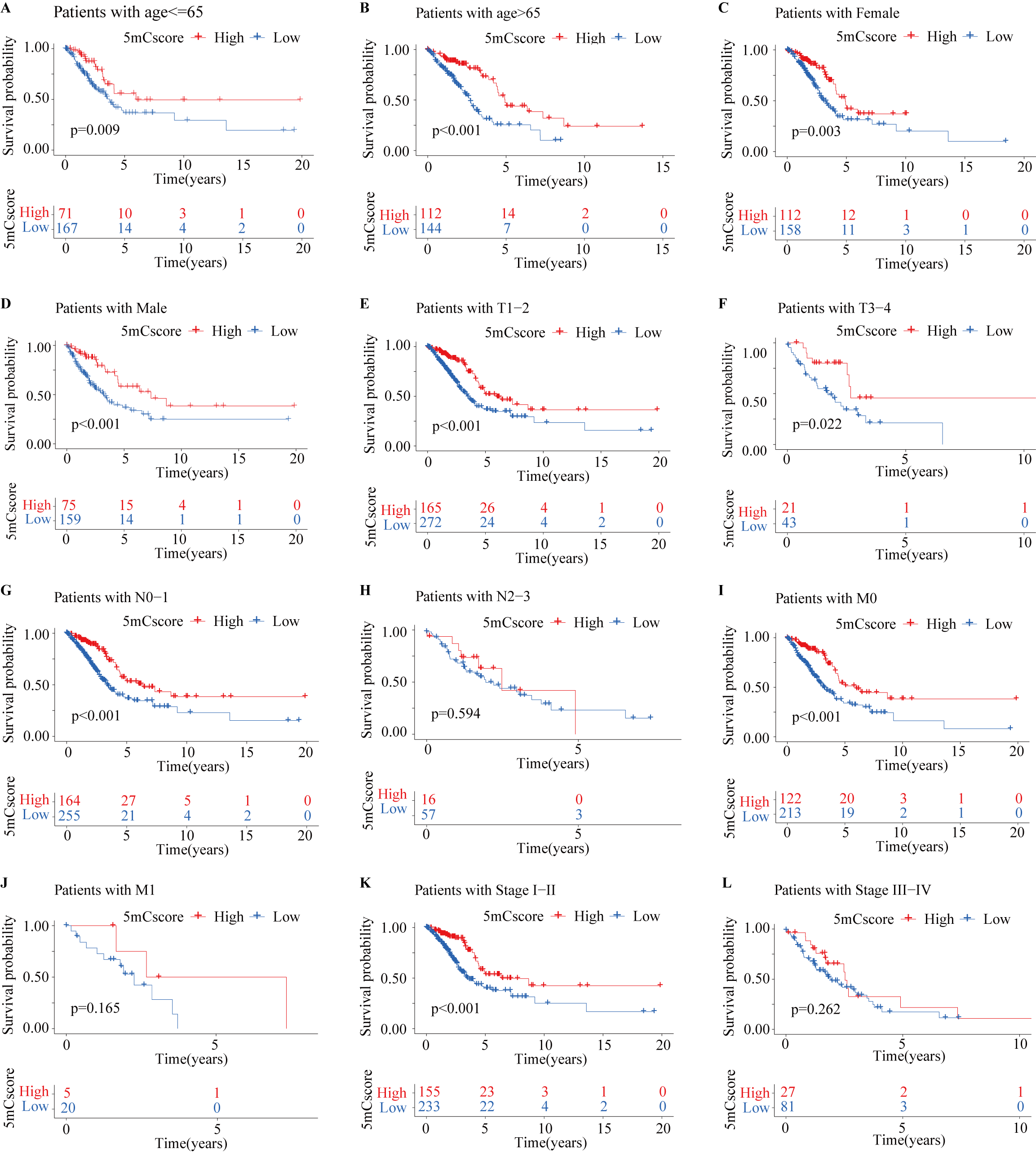

Supplement: Supplementary file 1 [file Image6.TIF]

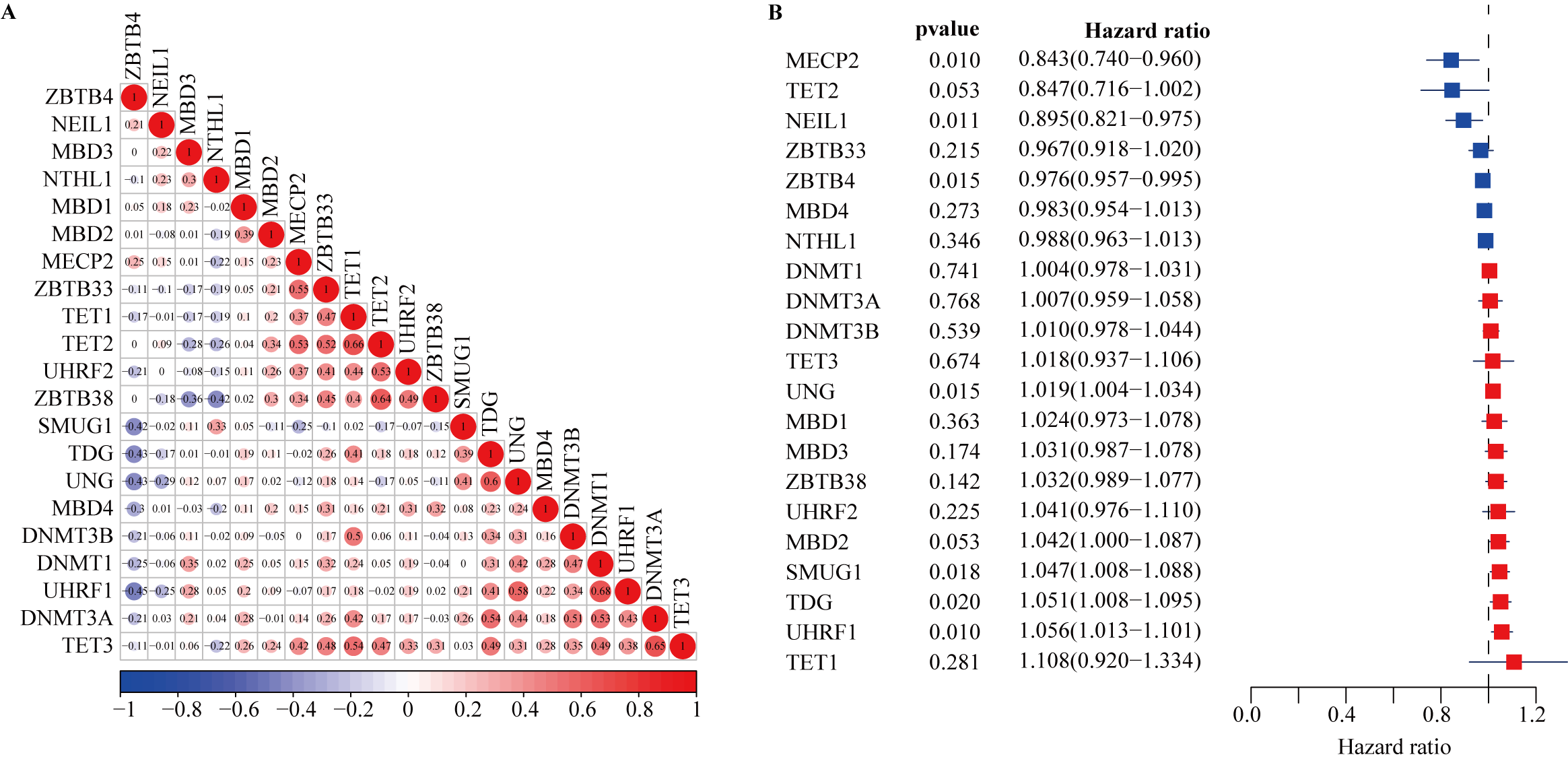

Supplement: Supplementary file 2 [file Image3.TIF]

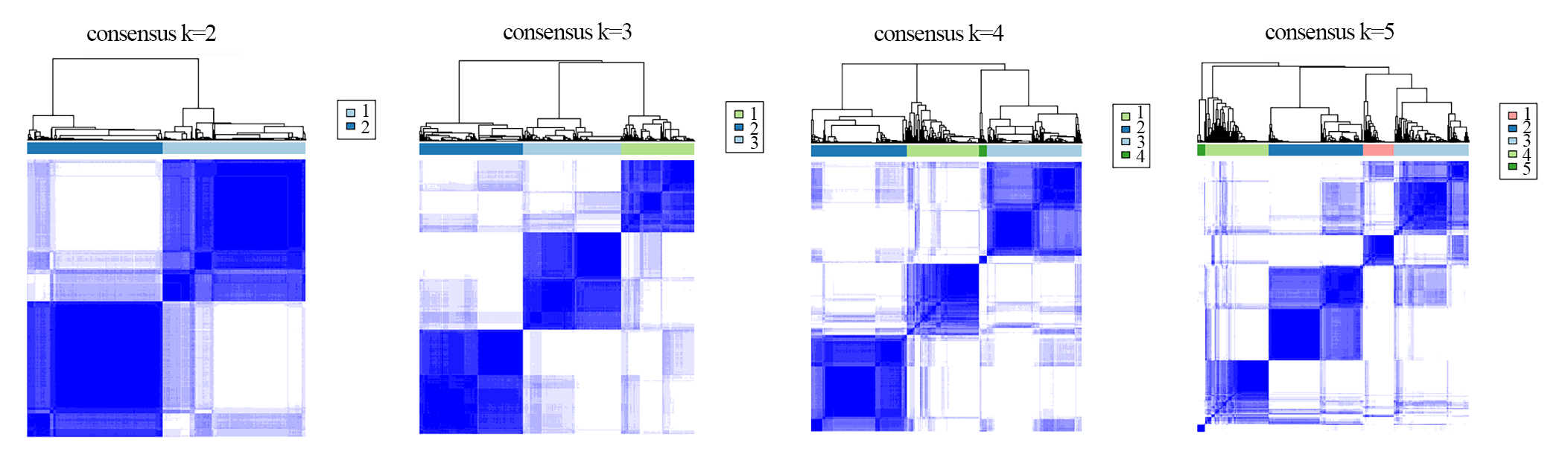

Supplement: Supplementary file 3 [file Image4.TIF]

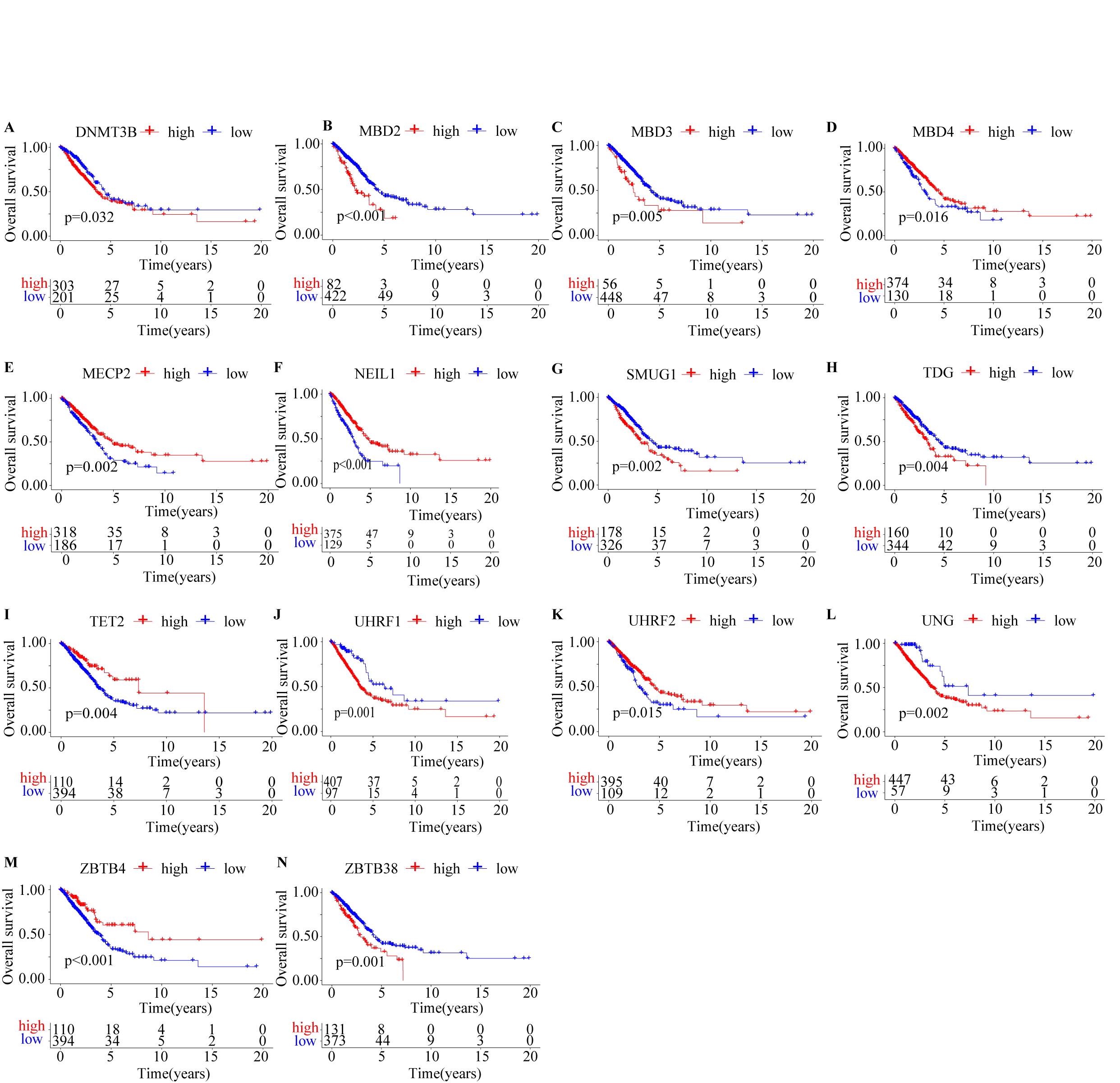

Supplement: Supplementary file 4 [file Image2.TIF]

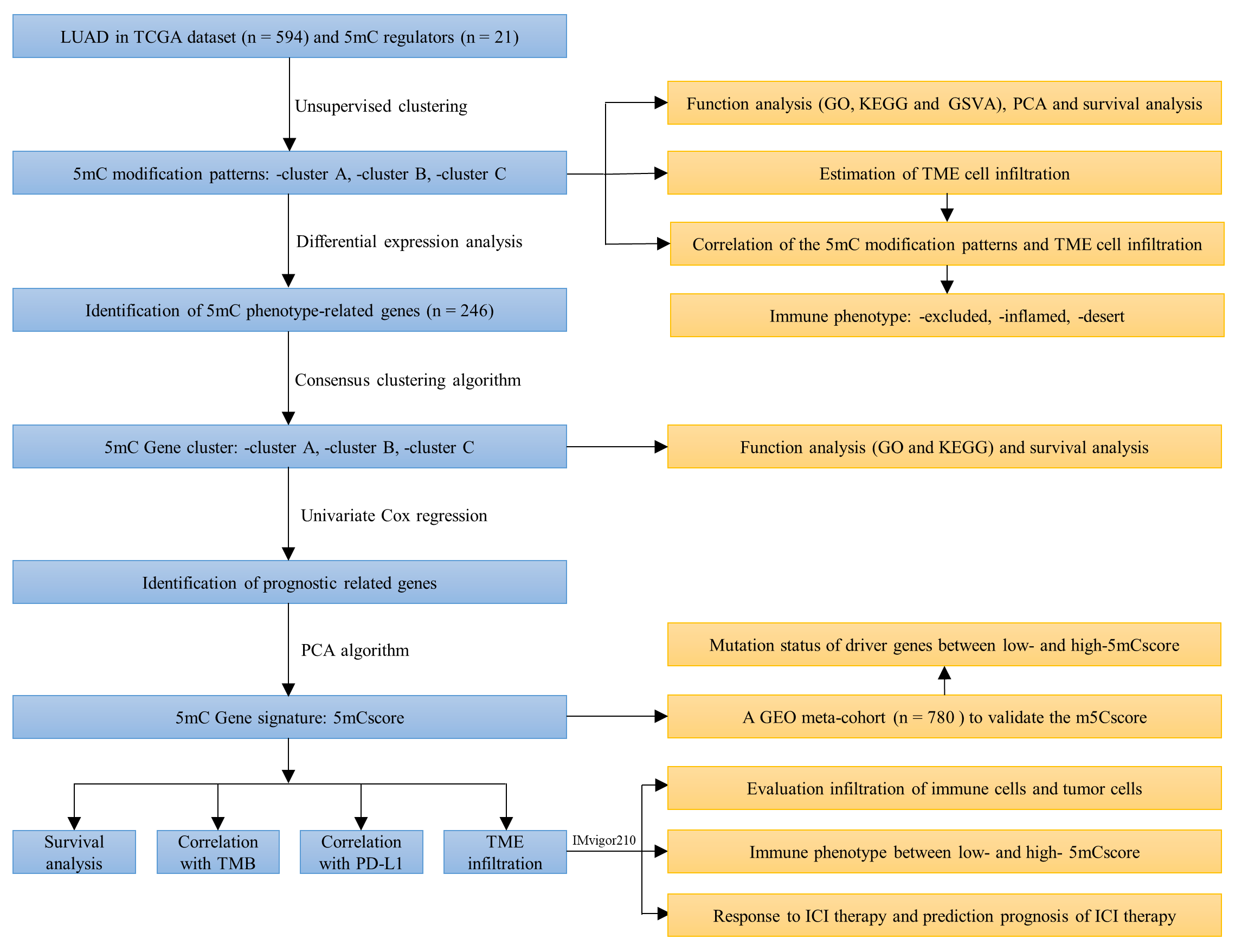

Supplement: Supplementary file 5 [file Image1.TIF]

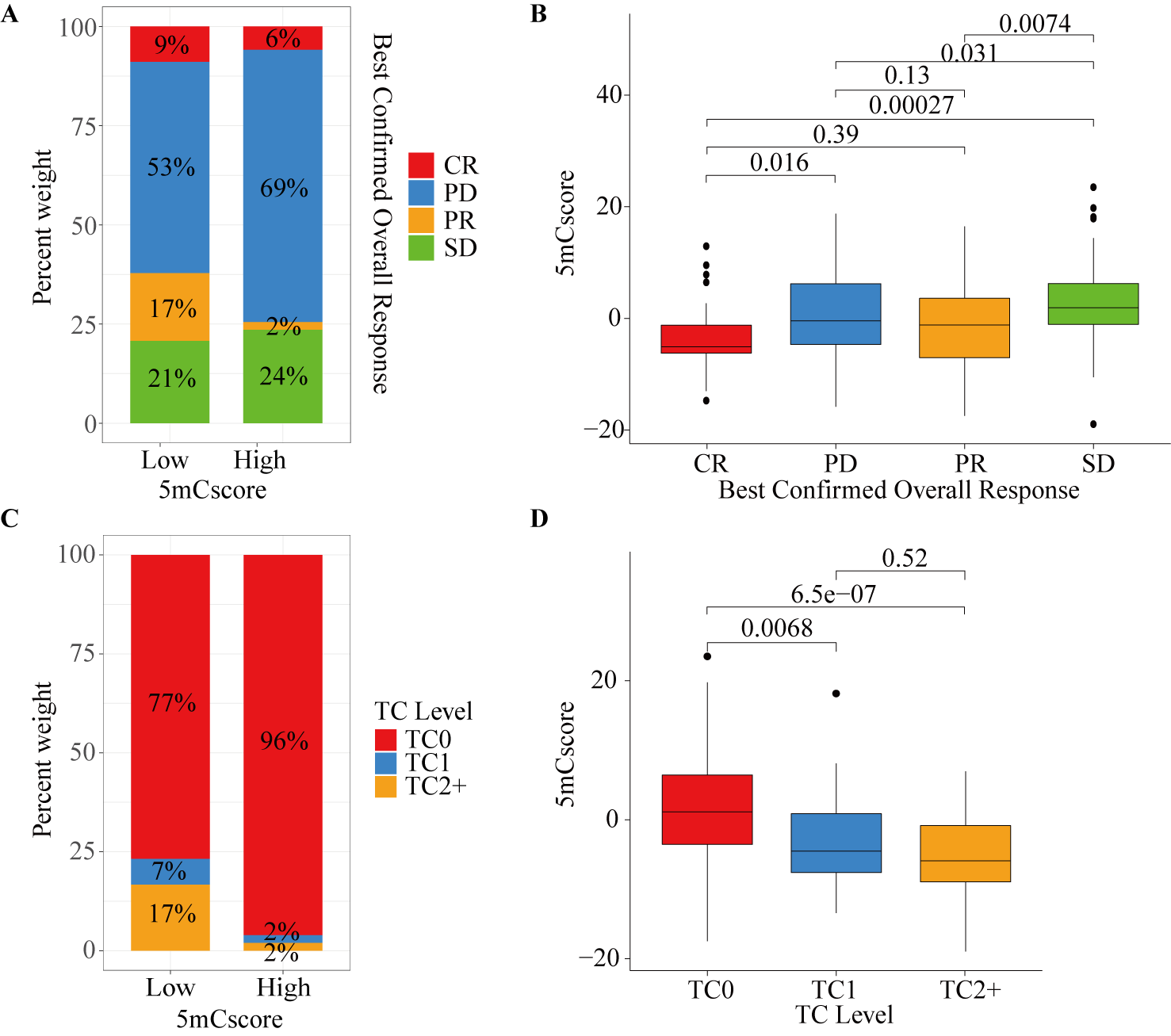

Supplement: Supplementary file 6 [file Image7.TIF]

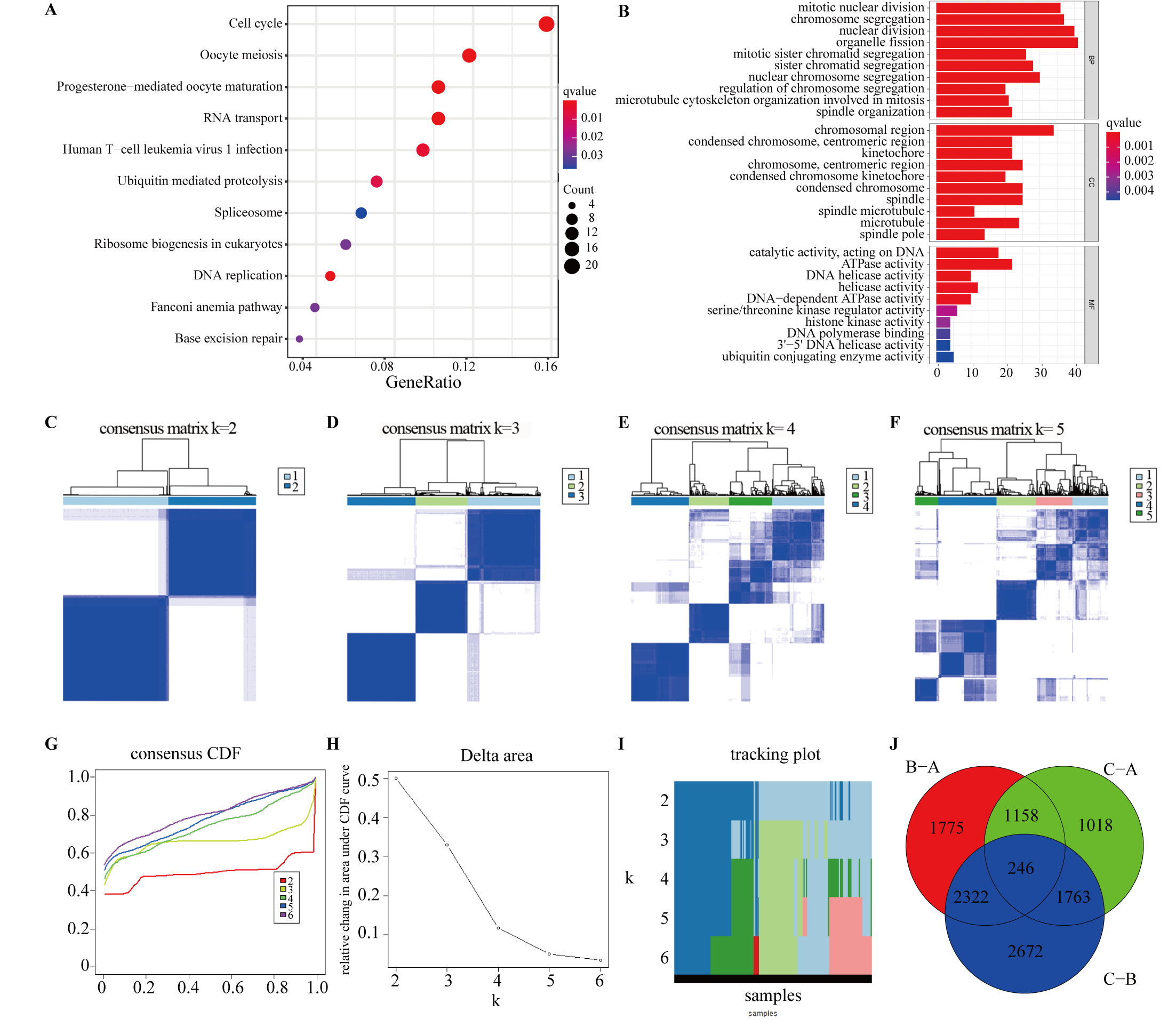

Supplement: Supplementary file 7 [file Image5.TIF]
